# Annexins and Membrane Repair Dysfunctions in Muscular Dystrophies

**DOI:** 10.3390/ijms22105276

**Published:** 2021-05-17

**Authors:** Coralie Croissant, Romain Carmeille, Charlotte Brévart, Anthony Bouter

**Affiliations:** Institute of Chemistry and Biology of Membranes and Nano-Objects, UMR 5248, CNRS, University of Bordeaux, IPB, F-33600 Pessac, France; coralie33400@free.fr (C.C.); carmeiller7@gmail.com (R.C.); charlotte.brevart@outlook.fr (C.B.)

**Keywords:** annexins, muscular dystrophy, membrane repair, skeletal muscle, genetic modifiers, LGMD, FSHD, DMD

## Abstract

Muscular dystrophies constitute a group of genetic disorders that cause weakness and progressive loss of skeletal muscle mass. Among them, Miyoshi muscular dystrophy 1 (MMD1), limb girdle muscular dystrophy type R2 (LGMDR2/2B), and LGMDR12 (2L) are characterized by mutation in gene encoding key membrane-repair protein, which leads to severe dysfunctions in sarcolemma repair. Cell membrane disruption is a physiological event induced by mechanical stress, such as muscle contraction and stretching. Like many eukaryotic cells, muscle fibers possess a protein machinery ensuring fast resealing of damaged plasma membrane. Members of the annexins A (ANXA) family belong to this protein machinery. ANXA are small soluble proteins, twelve in number in humans, which share the property of binding to membranes exposing negatively-charged phospholipids in the presence of calcium (Ca^2+^). Many ANXA have been reported to participate in membrane repair of varied cell types and species, including human skeletal muscle cells in which they may play a collective role in protection and repair of the sarcolemma. Here, we discuss the participation of ANXA in membrane repair of healthy skeletal muscle cells and how dysregulation of ANXA expression may impact the clinical severity of muscular dystrophies.

## 1. Introduction

Membrane ruptures induced by mechanical stress, such as contraction, stretching, or shearing, compromise cellular homeostasis and lead to cell death in the absence of fast resealing [1,2]. Frequency of cell membrane disruption is high in mammal tissues subjected to severe mechanical constraints, such as cardiac or skeletal muscle, epithelia, and endothelium [3,4,5,6]. For instance, adult rat muscle exposed to eccentric contractions exhibits about 20% of damaged myofibers, instead of 3.1% in basal conditions [5]. Healthy myofibers are able to repair disruptions of the sarcolemma (cell membrane of muscle cell) and survive: the rate of damaged myofibers dropped indeed to 5.6%, 24 h post-exercise [5]. How is skeletal muscle cell able to repair a damaged sarcolemma? This issue continues to be widely debated and the subject of intense research, even if the protein machinery starts being identified (see below). Discrepancies in experimental data have been indeed observed, which may result from the existence of different repair mechanisms depending on the cell type and the damage nature. While a membrane lesion less than 0.2 μm can be repaired passively, tension exerted by actin cytoskeleton on plasma membrane induces larger lesions and requires the intervention of active repair mechanisms [1,2]. These mechanisms are mostly triggered by the influx of extracellular calcium (Ca^2+^), which rushes into the cell through the membrane breach and invade the cytoplasm. The gradient of Ca^2+^ concentration created within the cytoplasm activates proteins of the membrane repair machinery, which then move towards the rupture site [7].

The absence of membrane repair causes the death of damaged cells and may contribute to tissue degeneration and the development of degenerative diseases [1]. A defective membrane repair machinery is observed in MMD1 [8,9], LGMDR1 (formerly 2A) [10], LGMDR2 (2B) [8], LGMD1C [11], and LGMDR12 (2L) (see chapter 4 for the nomenclature of LGMD) [12,13]. In addition, in certain forms of muscular dystrophy, such as Duchenne muscular dystrophy (DMD), the frequency of sarcolemma disruption is far higher than in normal muscle, which may lead to wear of the membrane repair machinery [5,14]. Here, we discuss the interplay between muscular dystrophies and sarcolemma repair and how dysregulations in ANXA expression may impact the severity of the disease.

## 2. Anatomy of Skeletal Muscle

**Figure 1:**
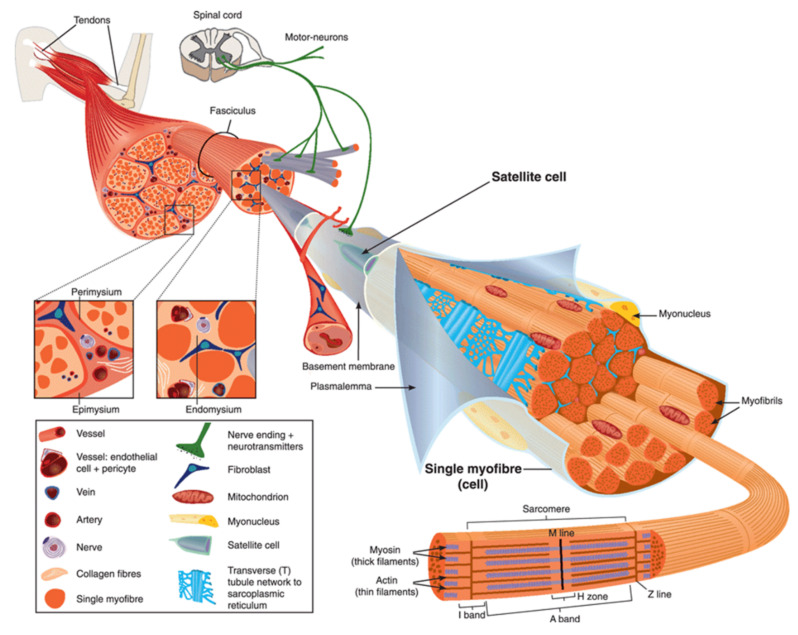
Scheme of skeletal muscle and associated structures. (**Left-hand part**) Epimysium, perimysium, and endomysium constitute three connective tissue layers that form the lattice network and associated basement membranes in which myofibers regenerate after injury. The epimysium is the outer layer that surrounds the entire muscle and is contiguous with the tendon and endosteum (fascia surrounding bone). The perimysium surrounds bundles of myofibers. The endomysium is located between individual muscle fibers. (**Right-hand part**) Satellite cells are located between basement membrane and sarcolemma. Sarcolemma bounds each myofiber, which is composed by multiple nuclei and the sarcoplasm that contains mitochondria, sarcoplasmic reticulum and myofibrils. The myofibril is the contractile unit of a myofiber. Specialized cytoskeleton within the myofibril forms repeated structures, called sarcomeres, which appear as a succession of light and dark bands under polarized light optical microscopy. The sarcoplasmic reticulum is the major provider of Ca^2+^ required for muscle contraction. It is connected to transverse tubules that surround sarcomeres. Adapted from Reference [15] with the Permission 5036470714502 from John Wiley and sons.

Human body is made up of approximately 650 muscles, with variability between individuals. Skeletal muscle tissue is itself made up of a collection of cell populations bounded by an envelope of fibrous connective tissue, called epimysium (Figure 1) [15]. This envelope ensures the maintenance and protection of the muscle during contraction, and is also the link between muscle and bones. A muscle is subdivided into a set of muscle bundles which are made up of several dozen muscle cells, all of which are surrounded by a connective sheath, called perimysium (Figure 1). Perimysium helps to structure the muscle and also to anchor muscle to bones at the level of tendons. The basal lamina or endomysium constitutes an extracellular matrix sheath that surrounds each muscle cell within a bundle (Figure 1). This extracellular matrix, essentially composed of collagen, ensures the stability of the muscle fiber by interaction with the intracellular cytoskeleton. It also ensures the cohesion of muscle bundle by connecting neighboring muscle cells. 

Skeletal muscle is made up of two main cell populations: skeletal muscle cells, also called muscle fibers or myofibers or myocytes, and mononuclear stem cells, called satellite cells (Figure 1). Satellite cells are generally quiescent, with the ability to proliferate, differentiate and fuse to form new myofibers. These cells are considered to be the main contributor to post-natal muscle growth and maintenance [16]. 

Myofibers are definitely special cells in terms of morphology and function. They exhibit a tubular and elongated shape and measure from a few hundred µm to several tens of centimeters in length for a diameter between 10 and 100 µm. Sarcolemma defines cytoplasm (sarcoplasm), which is composed by mitochondria, specialized endoplasmic (sarcoplasmic) reticulum, as well as a set of basic organelles, and a large number of nuclei located on the periphery of the cell. 

Sarcolemma is subjected to severe mechanical stress, more than in any other cell type, due to its huge surface and to contraction and stretching processes that it regularly undergoes [17]. To cope with these mechanical stresses, sarcolemma is supported by an exceptional protein framework, composed in particular of dystrophin, dystroglycans, and sarcoglycans [18]. Unrepaired sarcolemma damage leads to the death of myofiber, which is followed by inflammation, especially through the infiltration of macrophages, and regeneration phases, during which the satellite cells proliferate, differentiate and fuse to form a new fiber [19]. Satellite cells are not the only helping cells in skeletal muscle repair. Macrophages have been also reported to mediate sarcolemma repair through a mechanism involving dysferlin (DYSF) and phosphatidylserine (PS) [20]. DYSF accumulated at the membrane disruption site, subsequently to influx of Ca^2+^, promotes the accumulation of PS in the outer leaflet of the sarcolemma, which triggers the recruitment of macrophages. Excess of membrane and protein used during the resealing process and accumulated at the disruption site is engulfed by macrophages, which enables cell membrane integrity to be regained [20].

## 3. The Sarcolemma Repair Machinery

Studies of membrane repair-related processes are still the subject of intensive work, in particular to elucidate the mechanism(s) that take place depending on the cell type and the nature of the damage. Many reviews already exist on this topic [1,2,7,21,22,23]; here, we will focus on current understanding of membrane repair mechanisms in skeletal muscle cell.

Most observations of damaged myofibers, notably ours by transmission electron microscopy [24,25,26], have shown accumulation of lipid material at the site of membrane disruption, suggesting a repair process according to the “lipid patch” model proposed by P. McNeil in the late 1990s [27,28]. In this model, membrane resealing is based on the recruitment and accumulation of cytosolic vesicles. This process may be combined with an elongation of the sarcolemma [26], acting in concert to seal the rupture. The whole thing could be driven by a protein scaffold consisting of at least two parts, identified as the cap and shoulder subdomains [26,29].

### 3.1. The “Lipid Patch” Mechanism

**Figure 2:**
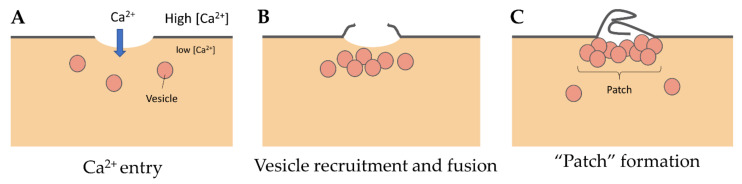
“Lipid patch” repair mechanism. (**A**) In the “lipid patch” model, the micrometer-sized rupture of the plasma membrane is followed by a massive entry of Ca^2+^ into the damaged cell. (**B**) The local increase in intracellular Ca^2+^ concentration initiates the recruitment of intracellular vesicles and (**C**) their fusion to form a vesicular aggregate or “patch”. The “patch” fuses at the ruptured area and plugs the lesion, which stops the mixing of extracellular and intracellular compartments.

Even if we do not know the precise nature and size of membrane damage occurring in exercised muscle, the uptake of albumin, whose estimated dimensions are 8 nm × 8 nm × 3 nm [30], observed into eccentrically injured myofibers suggests large sarcolemma lesion(s) of hundreds to thousands of square nanometers [5]. Such type of damage requires a rapid supply of lipid material to plug the rupture, one criterion which gave rise to the “lipid patch” hypothesis [27,28,31]. This model of membrane repair rests on early observations in sea urchin oocytes, where a fluorescent tracer injected intracellularly using a micropipette has been observed remaining sequestered in a membrane-rich area around the tip of the pipette [27]. Moreover, the same experiment carried out in the absence of Ca^2+^ has shown that the injected tracer diffused throughout the cell. In the “lipid patch” model, the rupture of the plasma membrane leads to a massive entry of extracellular Ca^2+^ into the cell (Figure 2A). Increase in the local concentration of free Ca^2+^ initiates the recruitment of intracellular vesicles (Figure 2B) which fuse to form a lipid aggregate or “patch” (Figure 2C) near the damaged membrane. The fusion of the “lipid patch” with the plasma membrane enables sealing of the rupture (Figure 2C). The “lipid patch”, thus, enables to stop the entry of Ca^2+^, which may induce apoptosis, or the release in the extracellular milieu of metabolites like fibroblast growth factor or creatine kinase, which is the main marker of muscular dystrophy [14]. The nature of the intracellular vesicles involved in the formation of the “lipid patch” is still debated. For sea urchin oocytes, the “patch” forms through homotypic fusion of organelles called Yolk granules [28]. This is probably the only known case of homotypic fusion. The implication of various types of vesicles or organelles has been reported in other cell types, including endosomes [32], enlargeosomes [33], mitochondria [34], and lysosomes [35,36,37]. Under conditions of intense stress leading to macro-rupture of the sarcolemma, it is likely that the formation of the “lipid patch” may involve all available intracellular vesicles, regardless of their nature. The “patch” might only serve as a transient barrier, which would then be gradually removed so that the sarcolemma regains its original composition and structure. For that purpose, recent investigations suggest the implication of helping cells, such as macrophages [20]. 

### 3.2. The Sarcolemma Repair Proteins 

Many proteins have been identified as important players in membrane repair, such as AHNAK, acid sphingomyelinase, ANXA, calpains, caveolins, ESCRT complexes, galactins, ferlins, Mitsugumin-53 (MG53), S100 proteins, SNAREs, or synaptotagmins [22,38,39]. In the section below, we will focus on membrane-repair proteins whose dysregulations are suspected of being involved in the development of muscular dystrophies (see Table 1 and Table 2).

#### 3.2.1. Dysferlin

Ferlins belong to a family of multiple C2-domain proteins identified in humans in the late 1990s [9,66,82]. They are found in most eukaryotes except in vascular plants, amoebae and fungi. In mammals, six members have been identified. These proteins present a large molecular weight (200–240 kDa), all contain a transmembrane domain in their C-Terminal part and five to seven C2 domains. This large and unusual number of C2 domains suggests evolutionary events of duplication. Ferlins are separated into two groups depending on the presence or absence of a DYSF domain [82]. Ferlin sequence mutations are associated with various pathologies, such as muscular dystrophies (DYSF), deafness syndromes (otoferlin, OTOF) in humans and mice, and infertility in Caenorhabditis elegans (Fer-1) and Drosophila melanogaster (misfire) [82]. 

**Figure 3:**
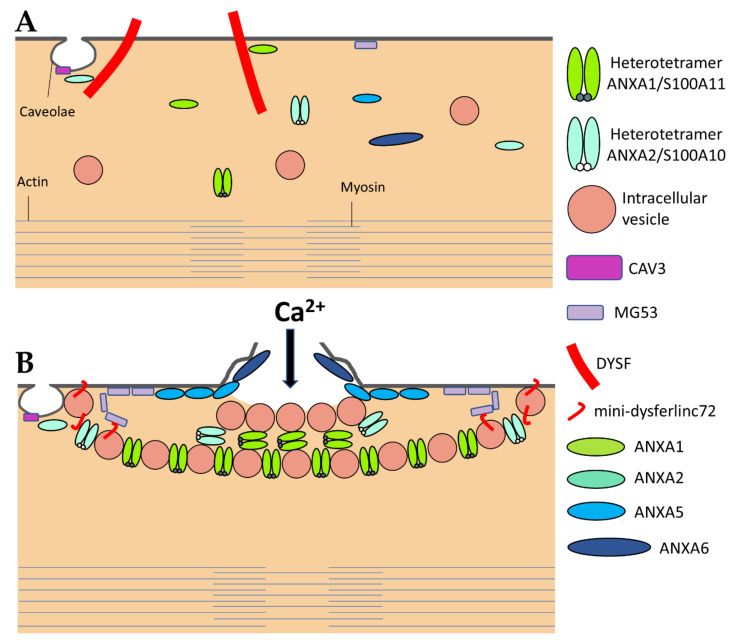
The sarcolemma repair machinery. (**A**) In normal, intact myofibers, full-length DYSF is localized at the sarcolemma where it interacts with ANXA1 and ANXA2. ANXA2 may also interact with CAV3. MG53 is mostly tethered to the sarcolemma. All other components of the membrane repair machinery may be soluble in the sarcoplasm. ANXA1 and A2 exist as both monomeric and heterotetrameric forms with their binding partners S100A10 (for ANXA2) and S100A11 (for ANXA1). (**B**) A disruption of the sarcolemma results in the influx of Ca^2+^ and oxidized milieu into the sarcoplasm that activates (1) the recruitment of ANXA5 and MG53 to the edges of the torn membrane that form arrays, which stabilize and prevent the expansion of the rupture, (2) the formation of the “lipid patch” through interaction of ANXA1 and ANXA2 -covered vesicles, (3) generation of mini-dysferlinec72 and the recruitment of mini-dysferlinec72-covered vesicles, that ensures hanging of the patch to the damaged sarcolemma, and (4) the recruitment of ANXA6 at the disruption site that may induce folding and curvature of the sarcolemma, which promote the formation of a tight membrane structure, i.e., the repair cap subdomain. Adapted from [24,26,40,41,46,55,70].

DYSF has been the first ferlin reported to be a key membrane-repair protein, which uses its fusogenic property to participate in membrane resealing [8]. The different C2 domains of DYSF can bind different kind of lipids found in biological membranes, including PS, phosphatidylinositol 4-phosphate((PtdIns(4)P), and phosphatidylinositol 4,5-bisphosphate (PtdIns(4,5)P(2)) [83]. The presence of Ca^2+^ increases affinity of DYSF for PS-containing membranes [84]. After sarcolemma rupture, increase in the local concentration of Ca^2+^ might, therefore, initiate the binding of DYSF to accessible either vesicular or plasma membranes (Figure 3) [46]. Interaction of C2 domains with membranes alters the structure of the bilayer by inducing curvature and clustering of negatively-charged phospholipids that might promote membrane fusion [67]. In human myotubes, a truncated DYSF, called mini-dysferlinec72, is recruited to the site of membrane injury instead of the full-length form [55]. This 72-kDa mini-dysferlinec72 is generated from DYSF through calpains-catalyzed cleavage, subsequent to an increase in the intracellular Ca^2+^ concentration. 

The recruitment of mini-dysferlinec72-covered vesicles has been shown to be pivotal for membrane repair [55,68,69]. DYSF present at the sarcolemma may be endocytosed within a minute after membrane damage [55]. Endocytosed DYSF might be then cleaved by calpains and recycled to the disruption site as mini-dysferlinec72, conveyed likely by intracellular vesicles (Figure 3). DYSF and mini-dysferlinec72 have been shown to interact with many components of the membrane repair machinery, including caveolin-3 (CAV3) [59], ANXA1 and A2 [40], and MG53 [70]. Cooperation between MG53 and mini-dysferlinec72 seems to be particularly pivotal for efficient membrane repair (Figure 3) [55,69]. 

#### 3.2.2. Caveolin-3

The caveolin family is made up of three members in humans, named caveolin-1 (CAV1), CAV2, and CAV3. Caveolins are small integral membrane proteins essential for the formation of caveolae, which are invaginated membrane structures 60 to 80 nm in diameter (Figure 3). Caveolae formation is ensured by the anchoring and oligomerization of caveolins to the plasma membrane, and by the interaction of caveolins with cavins [60]. CAV1 and CAV2 are expressed in a wide range of tissues including endothelial cells, adipocytes, fibroblasts and smooth-muscle cells. CAV3 is specifically expressed in skeletal and cardiac muscle, where it is the sole isoform present. The CAV3 sequence is composed of a horseshoe-shaped transmembrane region that enables membrane anchoring and a “Caveolin Scaffolding Domain” essential for caveolins oligomerization and interaction with other proteins [61]. Several functions are associated with caveolae, such as signaling pathways, endocytosis, regulation of lipid assimilation and storage, or the response to mechanical stress [60]. Muscle tissue is particularly rich in caveolae, whose flattening has been shown to protect the sarcolemma from mechanical damage by reducing tension exerted by the cortical cytoskeleton [62]. Flattening of caveolae is a rapid process, of the order of a minute, which is carried out by reversible dissociation of cavins with CAV3 [62]. Caveolae, therefore, seem to play a preventive role, limiting the appearance of rupture of the plasma membrane by controlling membrane tension. A direct involvement of CAV3 in sarcolemma repair has also been suggested but remains to be elucidated [63,64]. This role may be related to other key membrane-repair proteins, such as MG53 [65] or DYSF [59]. 

#### 3.2.3. Anoctamin-5

Anoctamin-5 (ANO5), also called Transmembrane protein 16E (TMEM16E), belongs to the TMEM16 family of proteins, which carry ten transmembrane regions with cytosolic N- and C-terminal extensions [85,86,87]. The TMEM16 family includes ion channels and phospholipid scramblases [86,88,89]. ANO5 is highly expressed in muscle and bone, in which it presents likely scramblase activity [90,91]. ANO5 carries a specific SCRD domain responsible for scrambling phospholipids [92]; nevertheless, this SCRD domain has been shown to be dispensable for membrane repair, suggesting a scramblase-independent role of ANO5 in membrane repair [50,51,52,53,54]. For instance, the R58W or R758C mutations in ANO5 lead to defective membrane repair, while both mutations are located outside the SCRD domain [53,54]. It has been observed that ANXA trafficking is abnormal in ANO5-KO fibers, suggesting that ANO5 may facilitate translocation of key repair proteins to the membrane disruption site [54].

#### 3.2.4. MG53

MG53 is a protein belonging to the tripartite motif (TRIM) protein superfamily. TRIM proteins are present in all metazoans, the number of isoforms varying greatly depending on the species (65 in humans) [93]. The TRIM superfamily is composed by eleven distinct families, which possess a highly conserved tripartite N-terminal domain called RBCC [93]. This tripartite motif is composed by a RING domain and 1 to 2 B-box domains (denoted B1 and B2), as well as, in most cases, a coiled-coil domain. The RING domain is a zinc binding domain responsible for Ubiquitin-E3 ligase activity. The B-box domain(s) are also zinc binding motifs, allowing interaction with other proteins [94]. Finally, the coiled-coil domain enables homo- or hetero-oligomerization of TRIM proteins. The different families of TRIM proteins are listed according to the motifs present in their C-terminal region. 

MG53, also known as TRIM72, is a 53-kDa protein specific for heart muscle and skeletal muscle [95]. Murine myofibers rendered deficient for MG53 are unable to reseal sarcolemma damage and MG53 null mice show the progressive development of myopathy [70]. Whereas most membrane repair proteins are triggered by increase of Ca^2+^ concentration, MG53 is activated through the entry of oxidized milieu from the extracellular space into the reduced cytoplasm. In resting conditions, MG53 is tethered to plasma membrane and intracellular vesicles. 

Upon sarcolemma injury, intracellular oxidation causes homo-oligomerization of MG53 monomers at the plasma membrane (Figure 3) [70]. Preliminary oligomerization of MG53 acts as a nucleation site for recruitment of MG53-tethered intracellular vesicles toward the injury site. Nucleation process rests mainly on disulfide bonds. MG53 oligomerization participates in the recruitment of vesicles to form a barrier facilitating membrane resealing. High Ca^2+^ concentration is, nevertheless, required to ensure vesicles aggregation as a final step of membrane resealing [70]. MG53 acts in membrane repair in collaboration with DYSF and CAV3 with which it interacts (Figure 3) [65]. It has, thus, been shown that the ectopic co-expression of the mutant P104L CAV3 leads to the retention of MG53 in the Golgi apparatus [65]. Injection of recombinant MG53 is foreseen as therapeutic treatment of muscular dystrophies. It has been indeed observed that injection of MG53 at a dose of 8 mg/kg twice a day for four days significantly decreases the proportion of damaged myofibers in DMD murine model [71].

#### 3.2.5. Calpains

Calpains form a large family of intracellular Ca^2+^-dependent proteases that are present in most eukaryotic cells and some bacteria [96]. Calpains share the presence of a CysPc domain containing a reactive cysteine. They are usually divided into two large groups (conventional and unconventional). Nine of the fifteen calpain human genes code for conventional calpains, which possess a C2-like domain, as well as an EF-hand-type Ca^2+^-binding domain [96]. They function as heterodimer by binding the catalytic subunit to the small regulatory subunit, called CAPNS1. Calpain-1 (CAPN1 or µ) and CAPN2 (or m) are expressed ubiquitously, while CAPN3 is found specifically in skeletal muscle. Calpains interact with major actors of membrane repair, such as DYSF and ANXA [55,56], and deficiency in CAPN3 gives rise to the most common LGMD, namely LGMDR1 (2A), which may result from defect in sarcomere remodeling [57]. During membrane injury, CAPN3 is responsible for the formation of mini-dysferlinec72 [55]. Meanwhile, CAPN1 and CAPN2 may play a role in membrane repair by promoting cytoskeletal remodeling notably by rapid degradation of vimentin and talin [58]. Their action on the cytoskeleton at the level of the membrane rupture area could also decrease surface tension of the plasma membrane and free up space for the repair machinery.

#### 3.2.6. Annexins

**Figure 4:**
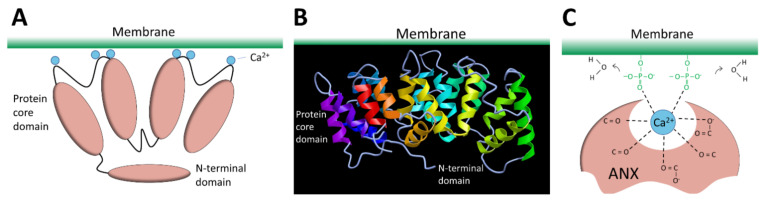
Structure of ANX. (**A**) Schematic representation of ANX. The four repeat sequences of the core domain fold into a curved-shape disk. The convex face binds Ca^2+^ (blue spheres) and interacts with membrane. The N-terminal domain varies between ANX and regulates their function by interacting with other proteins. (**B**) Representation of the tertiary structure of ANX. Image of 1AVH (human ANXA5) [103] created with MMDB [104]. (**C**) Schematic representation of molecular interactions between ANX, Ca^2+^, and membrane.

The ANX superfamily is composed of five major groups named A to E [97]. The vertebrate (A1–A13; A12 is unassigned) and invertebrate (B1–B14) ANX belong to the group A and B, respectively. In the C group are Fungi, molds, and close relative ANX (C1–C9). The groups D and E are composed by plants (D1–D20) and protists (E1–E20) ANX. ANX are characterized by a highly-conserved core domain composed of four repeat sequences (eight for ANXA6) [98] (Figure 4A). Each repeat is composed of seventy amino acids and contains five alpha-helical folded subdomains (Figure 4B). The membrane binding core has the shape of a slightly curved rhomboid with a convex membrane-binding face where Ca^2+^-binding loops are exposed (Figure 4A). The name “annexin” derives from the property of these proteins to “annex” membranes, “annex”, meaning “bring/hold together” in Greek. Binding of Ca^2+^ causes a conformational change in the protein, more prone to interact with membrane via electrostatic bridges between the Ca^2+^ ions and polar heads of membrane phospholipids [98]. In the absence of membrane, five of the seven Ca^2+^ coordination sites are provided by protein (carbonyl or carboxyl) oxygens and the remaining two coordination sites are occupied by water molecules. These two latter are replaced by phosphoryl moieties in membrane phospholipid-bound ANX (Figure 4C) [98,99]. The N-terminal end, variable in length and in sequence, protrudes from the concave side and faces the cytosol. It contains phosphorylation sites and binding sites for various molecular partners and is assumed to be responsible for the functional specificity of ANX. The binding of Ca^2+^ on ANX causes the deployment of this N-terminus that can then interact with other proteins, which, in turn, trigger their self-assembly or interaction with a third party, such as cytoskeleton [100]. ANXA constitute, therefore, an essential cog in the membrane repair machinery, by functioning as both membrane organizers and protein recruitment platforms [101,102].

##### ANXA1 et A2

ANXA1 and A2 were the first ANXA to be identified as participating in membrane repair [40]. With a molecular weight of 37 and 38kDa, respectively, ANXA1 and A2 share many features, including the ability to aggregate vesicles in the presence of Ca^2+^ [105,106,107] and to interact with S100 proteins [108]. Different models of membrane aggregation have been proposed. Monomeric ANXA1 may anchor a second membrane via a binding site located in the N-terminal domain [109]. A second mechanism proposes that dimerization of ANXA2 occurs via the N-terminal domain, enabling convex faces to interact with two adjacent membranes [110]. Finally, a third model involves the formation of ANXA/S100 hetero-tetrameric complex, with two peripherical ANXA and two central S100 proteins, enabling each ANXA to bind a membrane [106]. This ability to aggregate membranes, particularly intracellular vesicles, may play a role in the formation of the “lipid patch” (Figure 3). Reciprocally, S100 proteins are crucial for membrane repair [111]. In addition, ANXA1 and A2 interact with DYSF, forming a complex that is thought to be pivotal in the formation of the “lipid patch” [40] (Figure 3). ANXA1/S100A11 complex may be responsible for vesicles aggregation, yet ANXA2/S100A10 would mainly be in charge of recruiting the “lipid patch” to the disruption site through its interaction with DYSF (Figure 3) [41]. The ANXA2/S100A11 complex may also promote repolymerization of actin and facilitates formation of the new membrane [42,43].

##### ANXA4

ANXA4 is one of the smallest ANXA, with a molecular weight of 36kDa. Like ANXA1 and A2, ANXA4 is able to aggregate vesicles in the presence of Ca^2+^ [112]. Kaetzel and collaborators have observed that the distance between two aggregated membranes was compatible with two layers of ANXA4, suggesting possible homo-oligomerization [112]. Although not yet demonstrated, this result could indicate a role of ANXA4 in the recruitment of vesicles during membrane repair, as proposed for ANXA1 and A2. ANXA4 is also able to form trimers and two-dimensional (2D) arrays on artificial membranes [112], and to self-assemble on biological membranes to form immobile aggregates [113]. This property shared with ANXA5 could also intervene in membrane repair by stabilizing the damaged membrane (see below). Involvement of ANXA4 in membrane repair has been mainly studied in cancer cells, where accumulated as trimers at the damaged area it induces invagination of edges of the disruption site in order to facilitate constriction of the tear by ANXA6 [44]. As far as we know, involvement of ANXA4 in membrane repair of muscle cells has not been yet investigated.

##### ANXA5

ANXA5 is the smallest ANXA, with a molecular weight of 35kDa. In contrast to most ANXA, ANXA5 is unable to aggregate lipid vesicles [105]. Actually, the main feature of ANXA5 is the ability to create 2D arrays when interacting with a membrane in the presence of Ca^2+^ [114]. This property plays a crucial role in membrane repair by stabilizing the damaged membrane and preventing expansion of the tear [24,45]. We have revealed indeed that membrane repair in ANXA5-deficient muscle cells is rescued by the addition of recombinant wild-type ANXA5, but not by an ANXA5 mutant that lacks the ability of forming 2D arrays [24,45]. Through TEM observations, we have observed that ANXA5 binds mainly to the edges of the torn membrane and proposed that the formation of 2D arrays strengthen the membrane and prevents wound expansion [24] (Figure 3). 

##### ANXA6

ANXA6 is the largest ANXA due to the presence of two ANX core domains conferring a molecular weight of 68kDa [98]. At high concentration of Ca^2+^ (nearly 2 mM), ANXA6 is able to bind two distinct PS-containing membranes [115]. ANXA6 has also been reported to induce rearrangement of membrane structures [44,116]. Human or zebrafish muscle [26,46] and cancer [44] cells rendered deficient for ANXA6 suffer from a defect of membrane repair. In addition, ANXA6 knockdown in zebrafish leads to a form of myopathy [46]. In damaged skeletal muscle cells, ANXA6 is rapidly recruited to the disruption site by mainly interacting with the sarcolemma [26,29,46]. By inducing folding and curvature of the extensions of cell membrane, ANXA6 may allow to form a tight structure plugging the hole [26,29,46].

Once sarcolemma has been resealed, ANXA6 is exclusively found in the tight structure positioned on the exterior surface of the myofiber, termed the “repair cap” subdomain [26,29]. It may, therefore, help at condensing membranes to be eliminated, this elimination being probably performed by macrophages [20]. The presence of two ANX core domains gives to ANXA6 the ability to bridge two adjacent membranes, such as two regions of the cell and/or vesicle membranes, which promote membrane repair.

Injection of recombinant ANXA6 may be foreseen as therapeutic treatment of muscular dystrophies. It has been indeed observed that recombinant ANXA6 protected against acute muscle injury in a murine model of LGMDR5 (2C) [47].

##### ANXA7

ANXA7 was the first ANXA identified in humans [117]. In muscle cells, ANXA7 is present in two isoforms: a 47 kDa and a 51 kDa isoform, respectively, specific to myoblast and myofiber [118]. To date, involvement of ANXA7 in membrane repair has been reported only in cancer cells, where it enables the formation of the ESCRT-III complex via the recruitment of the ALG-2 and ALIX proteins to the damaged membrane [48]. Here, the ESCRT-III complex may induce the formation of microvesicles to eliminate the damage area. No study has reported the involvement of ANXA7 in membrane repair of muscle cells. However, the localization of ANXA7 has been shown to be altered in myofibers of patients with Duchenne muscular dystrophy [49]. Since ANXA7 is able to aggregate vesicles and promote membrane fusion during exocytosis in a Ca^2+^-dependent manner [117,119], its participation in the formation of the “lipid patch” is not excluded.

## 4. Aetiology and Nomenclature of Muscular Dystrophies

There are hundreds of different myopathies, all involving progressive weakness and degeneration of skeletal muscle. Most of them are devastating diseases leading to loss of ambulation, difficulties in breathing and eating and leading to premature death. Muscular dystrophies result from gene mutations leading to four main pathophysiological mechanisms: (1) instability of the sarcolemma (e.g., DMD), (2) repair defects of the sarcolemma (many LGMD), (3) senescence of satellite cells, and (4) disorganization of the sarcomere. 

LGMD, which encompass about thirty identified diseases in humans, may often result from a defect in the membrane repair process due to mutations in four main genes, namely DYS, CAV3, CAPN3, and ANO5 (Table 2). As the name suggests, girdle myopathies affect mainly muscles of shoulder and pelvic girdles, and some of them even affect also cardiac and respiratory muscles. To date, twenty-six forms have been characterized based on identification of genetic mutations responsible for the disease. The global prevalence of LGMDs is estimated between one to nine per million people. 

LGMDs were first separated into two groups according to the mode of inheritance: autosomal dominant forms or LGMD1, and autosomal recessive forms or LGMD2 [120]. Each LGMD subtype was characterized by mutations in a specific gene. For example, LGMD1C was an autosomal dominant form linked to mutations in the gene encoding CAV3, while LGMD2B was an autosomal recessive form linked to mutations in the gene encoding DYSF. In this nomenclature, the last letter corresponded to the chronology of discovery of the pathology. Unfortunately, many new recessive LGMDs enriched the classification up to the letter Z. Driven by the European Neuromuscular Center, a new classification and nomenclature of LGMDs have been established in 2017 in order to form more homogeneous groups of pathologies and to consider new identified LGMDs [77]. Information on the mode of transmission and chronology of discovery has been kept, with D or R (dominant or recessive, respectively) form associated to a number indicating the chronological order. For sake of clarity, when known, protein linked to the pathology has been added. For example, LGMD2B became LGMDR2 DYSF-related. On the other hand, the definition of an LGMD has been revised, excluding some pathologies and added new ones to the classification [77]. Thus, CAV3-related LGMD1C is no longer considered to be LGMD. With this new classification, five autosomal dominant and twenty-five autosomal recessive forms are now listed.

Within this Review, the new nomenclature of LGMD is used, and the former code is indicated in brackets.

## 5. Membrane Repair and Muscular Dystrophies

### 5.1. LGMDR2 Dysferlin-Related (LGMD2B)

DYSF was the first ferlin gene identified in humans during genetic studies to identify gene(s) responsible for the development of LGMDR2 (2B) and MMD1 [9,66], the two most common dysferlinopathies. The prevalence of dysferlinopathies is estimated at two cases per million people, with around twelve thousand cases worldwide. Beside MMD1 and LGMDR2 (2B), the other dysferlinopathies are classified as rare clinical pictures. These pathologies share many similarities, such as a very high serum creatine kinase level, the age of onset of symptoms (early adulthood), and the slow progression of the pathology, leading to the loss of walking several years after the onset of the disease. Cardiac or respiratory involvement is very exceptional in dysferlinopathies unlike many myopathies [121]. In patients suffering from dysferlinopathies, a large variety of mutations has been identified in the DYSF gene, which is located on chromosome 2p12-14 [74]. Mutations, which are distributed throughout the coding sequence without any hot-spot, lead to a complete or partial absence of DYSF in skeletal muscle and are at the origin of heterogeneous disorders.

DYSF has been one of the first identified key membrane-repair proteins, whose deficiency leads to a membrane repair defect and the development of muscular dystrophy [8]. Bansal and collaborators have shown that DYSF-knockout mice were unable to repair sarcolemma damage [8]. Accumulation of cytoplasmic vesicles in the cortical region of murine damaged myofibers suggested that the absence of DYSF prevented attachment of the “lipid patch” to the disrupted area. DYSF may act in membrane repair predominantly through mini-dysferlin isoforms [55], which explain why mutations leading genomic deletion in the first exons induce moderate forms of dysferlinopathies [75]. Transgenic expression of natural mini-dysferlin in dysferlinopathic mice restores the ability of skeletal muscle fibers to repair sarcolemma damage [75]. This confirms the important role of mini-dysferlin in membrane repair observed in vitro and paves the way for the development of gene therapy based on the expression of mini-dysferlins. 

### 5.2. LGMDD4 Calpain3-Related (LGMD1I) and LGMDR1 Calpain3-Related (LGMD2A)

Calpains have complex relationship with membrane repair and muscular dystrophies development. CAPN1 and CAPN2, which exhibit broad histological distribution, play pivotal roles in membrane repair [56], likely by cleaving DYSF into mini-dysferlinc72 [55]. Loss of CAPN1 and CAPN2 leads to the development of a form of severe muscular dystrophy in mice, which presents phenotypic similarities with dysferlinopathies [72]. Implication of CAPN3, the muscle specific calpain, in sarcolemma repair is not so obvious and remains controversial [56,73]. CAPN3 deficiency may be associated to abnormal mitochondria biogenesis and activity, leading to reduced repair ability of muscle fibers [73].

### 5.3. LGMDR12 Anoctamin5-Related (LGMD2L)

Mutations in ANO5 lead to the development of LGMDR12 (2L) and type 3 (non-DYSF) Miyoshi myopathy (MMD3) [51]. LGMDR12 (2L) phenotype is characterized by proximal weakness, with prominent asymmetrical quadriceps femoris and biceps brachii atrophy. The MMD3 phenotype is associated with distal weakness, of calf muscles in particular. Typically, diseases develop in adulthood (age 20–50) with proximal limb weakness, high serum creatine kinase levels, asymmetric muscle atrophy and weakness [51,76]. With the use of electron microscopy, multifocal sarcolemmal lesions have been observed in both phenotypes [51,76]. The phenotypic heterogeneity associated with ANO5 mutations is reminiscent of that observed with DYSF mutations responsible for LGMDR2 (2B) and MMD1. Phenotypic similarities between ANO5-myopathies and dysferlinopathies, lead to hypothesize that ANO5 may function in membrane repair comparable to DYSF. Nevertheless, it has been reported that ANO5 overexpression is unable to rescue the repair defect in DYSF-null mice [52]. Most mutations introduce premature termination codons in ANO5 and likely a translation-coupled nonsense-mediated RNA decay mechanism that may lead to their more rapid degradation, suggesting an underlining loss-of-function mechanism [51]. Loss of ANO5 leads to impaired muscle regeneration, with inability of the muscle to recover from injury by satellite cells fusion [12]. It leads also to a defect in repair of sarcolemma, which is unable to reseal damage induced by laser ablation [12]. The precise role played by ANO5 in membrane repair and the mechanism dysregulated in ANO5-myopathies remain to be elucidated.

### 5.4. Rippling Muscle Disease Caveolin-3 Related (LGMD1C)

Mutations in CAV3 gene, which is mapped on chromosome 3p25, lead to various forms of muscular dystrophies, grouped together under the term caveolinopathies. The predominant form is LGMD1C, which is no longer classified as limb girdle muscular dystrophy [11,77]. The other pathologies correspond to a benign distal myopathy called “Rippling Muscle Disease”, hyperCKemia, and a form of familial hypertrophic cardiomyopathy [78]. Mutations are distributed throughout the sequence encoding CAV3 and the same mutation can cause very different clinical manifestations, between individuals of different families or within the same family [79]. One common feature of CAV3 mutations is a significant decrease in the presence of CAV3 in the sarcolemma of myofibers, which results in a disorganization of the T-tubule network, as well as an alteration of cell signaling pathways [78]. For instance, studies on mutations, such as P104L, ∆TFT 63–65, R26Q, and T77K, have shown that a significant proportion of CAV3 forms aggregates that are segregated in membrane of Golgi apparatus [80,81]. A single allele gene mutation is sufficient to render unfunctional CAV3 in muscle fibers, suggesting that the mutant isoform is able to block the wild-type protein [78]. 

Skeletal muscle cells with mutated CAV3 (notably P104L) suffer from a defect of membrane repair [65]. Nevertheless, we do not know yet if caveolins, particularly CAV3, are involved directly in the repair complex [122] or operate as a driver for membrane-repair proteins, such as DYSF [59] and MG53 [65]. Some CAV3 mutations (R27Q, T64P) have indeed been shown to be associated with a decrease in the expression of DYSF [59] or with the retention of MG53 and DYSF in the Golgi apparatus [65]. Conversely, some mutations in the DYSF gene induce a decrease in the expression of CAV3 [123]. Finally, for patients carrying the P28L mutation, an increase in membrane tension and hypersensitivity to membrane ruptures have been observed, suggesting sarcolemma is more prone to damage [62]. 

### 5.5. Duchenne Muscular Dystrophy Dystrophin-Related

DMD is caused by mutations in the dystrophin gene, which participates in the stabilization of the sarcolemma and ensures its resistance to mechanical stress during contraction. DMD is, therefore, characterized by a great fragility of the sarcolemma, increasing the frequency of ruptures, which requires increased repair and regeneration capacities [69,124]. More than thirty genes belonging to the dystrophin-associated protein complex (DAPC) have been observed as implied in different forms of inherited muscular dystrophies, involving proteins of extracellular matrix, sarcolemma, cytoskeleton, or nuclear envelope [125].

## 6. ANXA and Muscular Dystrophies

In 2003, Bansal and collaborators revealed for the first time a direct link between a failure in membrane repair, caused by mutations in DYSF gene, and the development of a muscular dystrophy, e.g., LGMDR2 (2B) [8]. The fundamental role of ANXA in membrane repair questions their implication in the development of muscular dystrophies. In humans, no correlation has been made to date between muscular dystrophy and alteration of an ANXA gene. However, for the same genetic mutation, patients suffering from muscular dystrophy may exhibit significant differences in clinical signs. Symptoms may vary in nature, as well as in severity [79,126,127,128]. These observations led to the hypothesis that genetic modifiers may exist in muscular dystrophies, including ANXA.

ANXA1 and ANXA2 interact with DYSF to mediate sarcolemma repair [40] and both ANXA have been reported to be upregulated in Italian [129], American [130], or Australian patients [131] suffering from dysferlinopathies. Overexpression of these ANXA is likely an attempt to counteract the absence of DYSF and restore cell membrane repair ability. It has been revealed that excess of ANXA2 that leaks from injured myofibers activates muscle-resident fibro/adipogenic precursors that differentiate into adipocytes, which gradually replace dysferlin-deficient myofibers leading to muscle degeneration [132]. ANXA2 may act, therefore, as a modifying factor which strongly influences, in a negative way, clinical consequences of dysferlinopathies. Overexpression of ANXA2 is also observed in DMD, Becker muscular dystrophy or LMGDR12 and shedding of ANX-positive vesicles have been shown in ANO5-knockout myofibers (LMGDR12), suggesting these diseases may result from fibrotic or adipogenic replacement of myofibers [54,129]. Recently, an increase of 32% in the expression of ANXA2 has been also observed in a rat model of desminopathy [133]. 

ANXA1 and ANXA2 are susceptible to cleavage by calpains [134], which may be critical for their function in membrane repair [40,56,135]. In calpainopathies, such as LGMDR1 (2A) [10], therefore, it is expected that calpains deficiency may lead to misfunction of ANXA and impairment of membrane resealing. 

In addition, a loss of function of ANXA1 and ANXA6 is observed in LGMDR12 and DMD. In damaged ANO5-knockout myofibers (LGMDR12), accumulation of both ANXA is reduced, altering the tight repair cap structure [54]. In DMD, ANXA1 and ANXA6 present a reduced expression leading to exacerbated sarcolemmal injury and delayed repair cap formation due to overexpression of osteopontin [128]. 

The role played by ANXA6 as a genetic modifier of muscular dystrophies is definitely the most described. It has been reported that ANXA6 knockdown in a zebrafish model of dysferlinopathy reinforces the dystrophic phenotype [46]. In addition, a truncated form of ANXA6, named ANXA6N32, has been identified in Sgcg-null mouse, a model of LGMDR5 (2C) [136] and in dysferlinopathic mice [137]. ANXA6N32 dramatically impairs translocation of the full-length ANXA6 to the membrane disruption site, disrupts the protein scaffold that is pivotal for membrane resealing, and enhances muscular dystrophy [136,137]. 

Finally, ANXA7 has been also reported as disturbed in skeletal muscle from patients suffering from DMD and MDX mouse, whereas normal muscle contains specifically a 51-kDa ANXA7 isoform, dystrophic muscle exhibits the additional 47-kDa isoform, usually found in undifferentiated myoblasts [49,118]. During progression of the disease, ANXA7 is gradually retrieved in higher concentration in the serum of patients, suggesting the absence of membrane resealing of injured myofibers and the leak of ANXA7 [49]. If its participation in sarcolemma repair remains to be established, ANXA7 has been shown to mediate membrane repair in cancer cells by enabling assembly of the ESCRT-III complex [48]. 

Understanding how ANXA can modify the evolution of muscular dystrophies remains a huge project. In particular, most hitherto carried-out studies have used animal models and some differences may exist in the etiology and severity of muscular dystrophies between humans and animals. It will be, therefore, interesting in the near future to be able to explore these questions in human skeletal muscle cells.

## Figures and Tables

**Table 1:** Overview of proteins involved in sarcolemma repair.

Protein	Symbol	Function	Binding Partners	Ref
Annexin A1	ANXA1	The “lipid patch” formation	DYSF, S100A11	[40,41,42,43]
Annexin A2	ANXA2	The “lipid patch” formation	DYSF, S100A10	[40,41,42,43]
Annexin A4	ANXA4	To be established	To be identified	[44]
Annexin A5	ANXA5	Strengthening the damaged membrane	To be identified	[24,45]
Annexin A6	ANXA6	Condensing membranes at the disruption site	To be identified	[26,29,46,47]
Annexin A7	ANXA7	To be established	To be identified	[48,49]
Anoctamin-5	ANO5	To be established	To be identified	[50,51,52,53,54]
Calpains	CAPN1-3	The formation of mini-dysferlinc72	DYSF	[55,56,57,58]
Caveolin-3	CAV3	Controlling membrane tension	DYSF, MG53	[59,60,61,62,63,64,65]
Dysferlin	DYSF	Recruiting the “lipid patch”	ANXA1, ANXA2, Calpains, CAV3, MG53	[8,9,40,46,55,59,66,67,68,69,70]
Mitsugumin-53	MG53	Strengthening the damaged membrane and recruiting the “lipid patch”	DYSF, CAV3	[65,70,71]

**Table 2:** Overview of muscular dystrophies linked to deficiency in sarcolemma repair.

New Nomenclature	Former Nomenclature	MutatedGene	Protein	Ref
LGMD D4 Calpain3-related	LGMD1I	*CAPN3*	Calpain-3	[72,73]
LGMD R1 Calpain3-related	LGMD2A	*CAPN3*	Calpain-3	[72,73]
LGMD R2 Dysferlin-related	LGMD2B	*DYSF*	Dysferlin	[8,9,66,74,75]
LGMD R12 Anoctamin5-related	LGMD2L	*ANO5*	Anoctamin-5	[12,51,76]
Rippling muscle disease Caveolin3-related	LGMD1C	*CAV3*	Caveolin-3	[11,77,78,79,80,81]
MMD1 or Miyoshi myopathy	MMD1 or Miyoshi myopathy	*DYSF*	Dysferlin	[8,9,66,74,75]

## Data Availability

No new data were created or analyzed in this study. Data sharing is not applicable to this article.

## Abbreviations

ALIX: ALG-2 Interacting Protein X; ANO5, Anoctamin-5; ANXA, group A of the annexins family; ANXA1, Annexin-A1; ANXA2, Annexin-A2; ANXA5, Annexin-A5; ANXA6, Annexin-A6; Ca^2+^, calcium; CAV3, caveolin-3; CAPN, calpain; DMD, Duchenne Muscular Dystrophy; DYSF, dysferlin; ESCRT, the Endosomal Sorting Complex Required for Transport; LGMD, Limb Girdle Muscular Dystrophy; MG53, Mitsugumin-53; MMD, Miyoshi Muscular Dystrophy; OTOF, otoferlin; PS, phosphatidylserine; PtdIns(4)P, phosphatidylinositol 4-phosphate; PtdIns(4,5)P(2), phosphatidylinositol 4,5-bisphosphate; SCRD, scrambling domain; SNARE, Soluble N-ethylmaleimide-sensitive-factor Attachment protein Receptor; TRIM, tripartite motif.
